# Supplementary material for: Hepatotoxicity risk factors in HIV-infected MSM with HBV/HCV coinfections: A cohort study in Northwestern China
Source: PLoS One. 2025 Oct 13;20(10):e0333371. doi: 10.1371/journal.pone.0333371 (PMC12517527; doi:10.1371/journal.pone.0333371)
Supplement: Attachment — Submitted filename: Comment to Editorial 8.docx [file pone.0333371.s001.docx]

Comment to Editorial
The manuscript entitled “Hepatotoxicity risk factors in HIV-infected MSM with HBV/HCV coinfections: a cohort study in northwest China” addresses a significant public health issue with robust epidemiological data and clear clinical implications. I recommend acceptance with minor revisions. Some points merit clarification, particularly regarding methodological details and interpretation of hepatotoxicity findings. Please find my detailed comments below focusing on methods and results.

Commentary to Authors

Dear Dr. Xinyu Ma and colleagues,
Your manuscript “Hepatotoxicity risk factors in HIV-infected MSM with HBV/HCV coinfections: a cohort study in northwest China” offers important insights into the prevalence and impact of HBV and HCV coinfections on ART-related hepatotoxicity in a large MSM cohort. The study is methodologically sound with comprehensive data collection and appropriate analyses. After careful review, I suggest addressing the following minor issues to improve clarity and completeness:

Abstract and Title

1. The title and abstract accurately reflect the scope and main findings. However, consider briefly specifying the retrospective cohort design and inclusion criteria in the abstract methods section for clarity. Expanding slightly on the clinical relevance of the findings in the conclusion would strengthen the abstract.

Introduction
2. The introduction provides a solid background on HIV, HBV, and HCV epidemiology and coinfection risks in MSM. To further contextualize the study, a concise statement discussing gaps in hepatotoxicity data specifically among Chinese MSM on ART would enhance the rationale.

Materials and Methods
3. The retrospective cohort design and inclusion criteria are adequately described. Clarify details on data completeness and handling of missing data, especially for follow-up laboratory results. Also, please specify whether alcohol use or other hepatotoxic exposures were assessed or controlled for, as these could confound outcomes.

Results
4. Results are clearly presented with appropriate use of Kaplan-Meier and Cox regression analyses. The differentiation of hepatotoxicity risks by coinfection type and severity is valuable. Including a table summarizing multivariate hazard ratios for major predictors would improve accessibility.

Discussion
5. The discussion thoughtfully interprets findings in light of existing literature, emphasizing differential hepatotoxicity risks and potential mechanisms. Expanding the commentary on the unexpected protective association of lower hemoglobin and hepatotoxicity with possible explanations would add depth.

Conclusion
6. Conclusions are well supported by data emphasizing the importance of early HBV/HCV screening and liver function monitoring in MSM initiating ART.

Figures and Tables
7. Figures and tables are well formatted and informative. Ensure axis labels and legends are fully explanatory for international readers.

Overall Concerns
8. No scientific or ethical concerns identified. The study shows strong adherence to ethical standards with anonymity and approved protocols.

Competing Interests
9. Disclosures are complete and present no conflicts impacting study validity.

English Language Editing
10. The manuscript is written in clear, professional English. Minor stylistic polishing could enhance readability without substantive changes.

Thank you for your important contribution. I look forward to your revised manuscript addressing these minor comments.

Best regards,

I Ketut Agus Somia
Reviewer
