# Supplementary material for: Hepatotoxicity risk factors in HIV-infected MSM with HBV/HCV coinfections: A cohort study in Northwestern China
Source: PLoS One. 2025 Oct 13;20(10):e0333371. doi: 10.1371/journal.pone.0333371 (PMC12517527; doi:10.1371/journal.pone.0333371)
Supplement: Attachment — Submitted filename: Response to Reviewers.docx [file pone.0333371.s003.docx]

**Response to Editor’s Comment:**

1.clearly state and justify what type of incidence (cumulative incidence or incidence rate) was measured.

Response:

Thank you for your thoughtful comment. We measured hepatotoxicity as an incidence rate rather than cumulative incidence. This decision was based on the fact that our study involved varying follow-up times for participants and included patients who were lost to follow-up. Specifically, the hepatotoxicity incidence rate was calculated as the number of new hepatotoxicity events per 1,000 person-years, which accurately reflects the risk over time for each participant, regardless of differences in follow-up duration. This method is particularly appropriate when dealing with censored data, as it accounts for both event occurrence and the time at risk for each individual. Thus, the measure used in our study is the incidence rate, as indicated by our calculation of new events per person-time.

Specifically, we have clarified this in the Methods section. The revised text now reads:

*"Hepatotoxicity incidence and mortality rates were calculated as the number of new hepatotoxicity events and deaths per 1,000 person-years (py) of follow-up. For the incidence rate, hepatotoxicity events were counted annually, and the rate was determined by dividing the number of events by the total person-years at risk, then multiplying by 1,000."*

2.clearly define hepatotoxicity in the methods section of the abstract

Response:

Thank you for your insightful comment. In response to your suggestion, we have added a brief definition of hepatotoxicity in the Methods section of the Abstract. While a detailed definition of hepatotoxicity, including the grading system based on the National Cancer Institute (NCI) Common Terminology Criteria for Adverse Events (CTCAE), is provided in the main manuscript, we have now included a concise description in the Abstract for clarity.

The revision in the Abstract now reads as follows:

*" Hepatotoxicity was classified into grades 0 (normal) to 4 (life-threatening) according to the degree of elevation in liver enzymes (AST or ALT) ."*

This revision ensures that the key concept of hepatotoxicity is clearly stated in the Abstract while maintaining the detailed explanation in the Methods section of the main manuscript.

3.to revise the abstract's results section so that the main findings are aligned with the study objectives

Response:

Thank you for your helpful feedback. In response to your suggestion, we have revised the Results section of the Abstract to ensure it is closely aligned with the study objectives. The revised version emphasizes the prevalence of HBV and HCV coinfections, their significant impact on hepatotoxicity risk, particularly grade ≥3 events, and the clinical factors identified as risk or protective determinants.

The revised Results section now reads as follows:

*"Among 4,690 HIV-infected MSM, the prevalence of HIV/HBV and HIV/HCV coinfections was 5.18% and 2.17%, respectively. Coinfected individuals had significantly elevated hepatotoxicity risks. HIV/HBV coinfection substantially increased the risk of any-grade hepatotoxicity (Hazard Ratio [HR]: 1.190, 95% Confidence Interval [CI]: 1.029–1.375) and grade ≥3 hepatotoxicity (HR: 3.161, 95% CI: 2.095–4.769). HIV/HCV coinfection was also associated with a higher risk of any-grade hepatotoxicity (HR: 1.311, 95% CI: 1.050–1.636). Older age at ART initiation, a shorter diagnosis-to-treatment interval, and HBV/HCV coinfection were identified as risk factors, while higher CD4+ T lymphocyte counts and lower hemoglobin levels were protective factors."*

4.the key words to include the setting/place in which the study was conducted

Response:

Thank you for your helpful suggestion. In response to your request, we have updated the Keywords section of the manuscript to include "Northwestern China", which reflects the study's geographic setting. The revised Keywords now read as follows:

*"HIV, HBV, HCV, MSM, antiretroviral therapy, hepatotoxicity, risk factors, cohort study, Northwestern China"*

**Response to Journal Requirements:**

1. Please ensure that your manuscript meets PLOS ONE's style requirements, including those for file naming.

Response:

We sincerely thank the editors for their careful review and helpful guidance. We have carefully revised the manuscript to ensure that it fully complies with PLOS ONE’s style requirements. Specifically, we have reformatted the text, references, tables, and figures according to the PLOS ONE formatting guidelines, and we have updated the file names to meet the journal’s specifications. The revised submission follows the templates provided in the links. In addition, we have added sections on Data Availability, Funding, and Competing Interests at the end of the manuscript as per the journal’s requirements.

2. We note that you have indicated that there are restrictions to data sharing for this study. For studies involving human research participant data or other sensitive data, we encourage authors to share de-identified or anonymized data. However, when data cannot be publicly shared for ethical reasons, we allow authors to make their data sets available upon request. Please update your Data Availability statement in the submission form accordingly.

Response:

Thank you for your guidance. We have updated the Data availability statement in the submission manuscript accordingly. The revised Data availability statement section now reads as follows:

*"The data underlying this study were obtained from the electronic medical record system of Xi’an Eighth Hospital and contain potentially identifying and sensitive patient information, including demographic and clinical follow-up data. Due to ethical and legal restrictions imposed by the Medical Ethics Committee of Xi’an Eighth Hospital, these data cannot be shared publicly. Researchers who meet the criteria for access to confidential data may request access by contacting the Medical Ethics Committee of Xi’an Eighth Hospital at bygcpll2018@163.com."*

3. Please review your reference list to ensure that it is complete and correct. If you have cited papers that have been retracted, please include the rationale for doing so in the manuscript text, or remove these references and replace them with relevant current references. Any changes to the reference list should be mentioned in the rebuttal letter that accompanies your revised manuscript. If you need to cite a retracted article, indicate the article’s retracted status in the References list and also include a citation and full reference for the retraction notice.

Response:

We thank the Editors for their guidance. We have updated the first reference of the original manuscript to ensure that the data source is up-to-date. The first reference has been updated from *"1. Cheruvu S and Marks K. Talal AH. Understanding the pathogenesis and management of hepatitis B/HIV and hepatitis B/hepatitis C virus coinfection. Clin Liver Dis. 2007; 11 (4) : 917-43, ix - x. doi: 10.1016 / j.carol carroll ld. 2007.08.007. PubMed PMID: 17981235."* to *" 1. UNICEF. Global and regional trends 2025 [updated July 2025; cited 2025 August 22nd]. Available from: https://data.unicef.org/topic/hivaids/global-regional-trends/."*

In the previous version, Reference #3 was incorrectly cited as *"3. Greenleaf A, Nhlabatsi B, Mahlalela K, Masangane Z, Ndlangamandla M, Philips N, et al. DYnamics of Contraception in Eswatini (DYCE): protocol for a high-frequency, longitudinal cell phone survey. BMJ Open. 2024;14(11):e090686. Epub 20241114. doi: 10.1136/bmjopen-2024-090686. PubMed PMID: 39542494; PubMed Central PMCID: PMCPMC11575318.*"

This has now been corrected to the appropriate reference:

*"3. Cai C, Tang H, Li D, Qin Q, Chen F, Jin Y, et al. Evolution of HIV Epidemic and Emerging Challenges - China, 1989-2023. China CDC Wkly. 2024;6(48):1251-6. doi: 10.46234/ccdcw2024.251. PubMed PMID: 39698324; PubMed Central PMCID: PMC11649988."*

In addition, three new references (now listed as #35 - #37 in the revised manuscript and also shown below) have been added in the Discussion section to support our arguments.

*"35.	Takano H, Yamaguchi J-I, Kato S, Hamada M, Tada M, Endo H. Downregulation of CYP1A2, CYP2B6, and CYP3A4 in Human Hepatocytes by Prolyl Hydroxylase Domain 2 Inhibitors via Hypoxia-Inducible Factor-α Stabilization. Drug Metab Dispos. 2021;49(1):20-30. doi: 10.1124/dmd.120.000124. PubMed PMID: 33087449.*

*36.	Wang Q, Liu G, Duan Y, Duo D, Zhu J, Li X. Exploring cytochrome P450 under hypoxia: potential pharmacological significance in drug metabolism and protection against high-altitude diseases. Drug Metab Dispos. 2025;53(2):100026. doi: 10.1016/j.dmd.2024.100026. PubMed PMID: 40023572.*

*37.	HHS. Guidelines for the Use of Antiretroviral Agents in Adults and Adolescents with HIV 2024 [updated September 12, 2024; cited 2025 August 19th]. Available from: https://clinicalinfo.hiv.gov/en/guidelines/hiv-clinical-guidelines-adult-and-adolescent-arv/whats-new."*

All changes to the reference list have been incorporated into the revised manuscript.

**Response to Reviewesrs' comments:**

**Reviewer #1:**

Abstract and Title

1. The title and abstract accurately reflect the scope and main findings. However, consider briefly specifying the retrospective cohort design and inclusion criteria in the abstract methods section for clarity. Expanding slightly on the clinical relevance of the findings in the conclusion would strengthen the abstract.

Response:

Thank you for your constructive feedback. In response to your suggestion, we have revised the Methods section of the Abstract to explicitly state the retrospective cohort design and to highlight the inclusion criteria for clarity.

The revised Methods section now reads:

*“This retrospective cohort study analyzed MSM who were newly diagnosed with HIV and initiated ART in Northwestern China between January 1st, 2005, and June 30th, 2019. A total of 4,690 MSM aged ≥18 years were included and categorized into three groups based on HBV or HCV coinfection status: HIV monoinfection, HIV/HBV coinfection, and HIV/HCV coinfection. Hepatotoxicity was classified into grades 0 (normal) to 4 (life-threatening) according to the degree of elevation in liver enzymes (AST or ALT) . Kaplan-Meier curves were used to evaluate the incidence of hepatotoxicity, mortality, and ART failure rates, while Cox proportional hazards models assessed the independent impact of coinfections on hepatotoxicity risk.”*

In response to your comment, we have revised the Conclusion section of the abstract to further emphasize the clinical relevance of our findings. Specifically, we have highlighted the increased risk of hepatotoxicity in HIV-infected MSM with HBV and HCV coinfections and emphasized the importance of early screening, regular liver function monitoring, and standardized antiviral therapy in HIV care. The revised Conclusion now reads as follows:

*"HIV/HBV and HIV/HCV coinfections significantly increased the risk of hepatotoxicity in HIV-infected MSM receiving ART. These findings underscore the importance of vigilant liver function monitoring in coinfected patients on ART, particularly in consideration of baseline factors such as age, time to treatment, CD4+ T-cell count, and hemoglobin level, to minimize interruptions and optimize outcomes."*

Introduction

2. The introduction provides a solid background on HIV, HBV, and HCV epidemiology and coinfection risks in MSM. To further contextualize the study, a concise statement discussing gaps in hepatotoxicity data specifically among Chinese MSM on ART would enhance the rationale.

Response:

Thank you for your valuable suggestion. In response to your comment, we have revised the Introduction to briefly highlight the paucity of data on ART-related hepatotoxicity in the MSM population in China, particularly in relation to HBV/HCV coinfection. This addition strengthens the rationale and underscores the significance of our study in addressing this gap. The revised text now reads as follows:

*“While ART has substantially improved patient outcomes, hepatotoxicity remains a major concern. This risk is particularly pronounced in individuals coinfected with HBV or HCV, whose prevalence continues to rise among MSM in China. However, few studies have systematically investigated ART-related hepatotoxicity and its risk factors in this key population, and data from Northwestern China are especially scarce.”*

Materials and Methods

3. *The retrospective cohort design and inclusion criteria are adequately described. Clarify details on data completeness and handling of missing data, especially for follow-up laboratory results. Also, please specify whether alcohol use or other hepatotoxic exposures were assessed or controlled for, as these could confound outcomes.*

Response:

Thank you for your valuable suggestion. In response to your comment, we have added details regarding data completeness, handling of missing data, and potential hepatotoxic exposures.

Data completeness and missing data:

We clarified that cases with missing baseline or follow-up laboratory results were excluded from the analysis. For other variables with missing values, no imputation was performed; analyses were conducted using available case data.

Potential hepatotoxic exposures:

We also specified that information on alcohol use and other hepatotoxic exposures (e.g., hepatotoxic medications) was not systematically recorded in the medical records, and therefore could not be adjusted for in our analyses. This limitation has been acknowledged in the revised manuscript.

The revisions have been incorporated into the Data sources and collection and Statistical analysis sections of the Materials and Methods. The revised text reads:

Data sources and collection:

*“Data completeness was assessed for all variables, particularly follow-up laboratory results (AST and ALT). In cases where baseline laboratory results or follow-up laboratory data were missing, these cases were excluded from the analysis. For any missing values in variables, no imputation was performed, and the analysis was conducted using available case data.”*

Statistical analysis:

“*Information on alcohol consumption and other potential hepatotoxic exposures (such as the use of hepatotoxic medications) was not systematically collected in the medical records; therefore, these factors were not controlled for in the analysis, which is a limitation of the study.”*

Results

4. Results are clearly presented with appropriate use of Kaplan-Meier and Cox regression analyses. The differentiation of hepatotoxicity risks by coinfection type and severity is valuable. Including a table summarizing multivariate hazard ratios for major predictors would improve accessibility.

Response:

Thank you for your helpful suggestion. In response to your comment, we have added the following sentence to the Results section to explicitly reference Table 4, which already provides the requested information regarding the multivariate hazard ratios (HR) and 95% confidence intervals (CI) for the major predictors of hepatotoxicity:

*“Table 4 summarizes the multivariate HR for the major predictors of hepatotoxicity and their 95% CI.”*

This addition clarifies that Table 4 already includes the necessary information, and we believe it enhances the accessibility and clarity of the results.

Discussion

5. The discussion thoughtfully interprets findings in light of existing literature, emphasizing differential hepatotoxicity risks and potential mechanisms. Expanding the commentary on the unexpected protective association of lower hemoglobin and hepatotoxicity with possible explanations would add depth.

Response:

Thank you for your thoughtful suggestion. In response, we have expanded the Discussion to provide a more detailed and evidence-based interpretation of the unexpected protective association between lower hemoglobin levels and hepatotoxicity. We have introduced several potential mechanisms, including the effects of reduced oxygenation, systemic inflammation, and altered pharmacokinetics, to explain this association. In addition, we have deleted the original, more ambiguous explanation and replaced it with a more comprehensive discussion. The revised discussion now reads as follows:

*“A significant protective association was observed between lower hemoglobin levels and hepatotoxicity, an unexpected finding that warrants further exploration. One possible explanation is that reduced hemoglobin may reflect a hypoxic state, which activates the HIF pathway and subsequently downregulates major hepatic cytochrome P450 enzymes, leading to decreased drug metabolic activation and attenuated hepatocellular injury. While this hypothesis provides a plausible explanation, further studies are needed to confirm the mechanism and clarify its clinical implications.*

Conclusion

6. Conclusions are well supported by data emphasizing the importance of early HBV/HCV screening and liver function monitoring in MSM initiating ART.

*We sincerely thank the Editor for the positive comment. We are pleased that you found our conclusions to be well supported by the data, particularly regarding the importance of early HBV/HCV screening and liver function monitoring in MSM initiating ART.*

Figures and Tables

7. Figures and tables are well formatted and informative. Ensure axis labels and legends are fully explanatory for international readers.

Response:

We sincerely appreciate your valuable suggestion regarding the clarity of axis labels and legends for international readers. In accordance with your advice, we have carefully reviewed all figures and tables to ensure that abbreviations are either avoided or explicitly defined.

Specifically, in Table 2, the abbreviation *“WBC”* has been replaced with *“White blood cell counts,”* *“Platelet”* has been replaced with *“Platelet counts,”* *“T.BIL”* has been replaced with *“Serum total bilirubin,”* and *“Scr”* has been replaced with *“Serum creatinine.”* In Figure 2, the y-axis label in panel (e) has been revised from *“WBC”* to *“White blood cell counts,”* and the label in panel (f) has been changed from *“Platelet”* to *“Platelet counts.”* Additionally, definitions for AST (Aspartate aminotransferase) and ALT (Alanine aminotransferase) have been added in the figure legend for Figure 2 to improve clarity.

The modified Figure 2 and its legend are as follows:

*Fig 2. Comparison of baseline characteristics of MSM grouped by infection status, including (a) age, (b) age at ART initiation, (c) interval between diagnosis and treatment, (d) CD4+ lymphocyte counts, (e) White blood cell counts, (f) platelet counts, (g) AST, (h) ALT, (i) marital status. Statistically significant pairwise comparison results are indicated as follows: a single asterisk (*) for 0.01 < P < 0.05, double asterisks (**) for 0.001 < P < 0.01, and triple asterisks (***) for P < 0.001. MSM, men who have sex with men; ART, antiretroviral therapy; AST,aspartate aminotransferase; ALT,alanine aminotransferase.*

In addition, all abbreviations appearing in the figures and tables of the manuscript have been defined in the figure legends, ensuring consistency with academic writing standards and guidelines.

We believe these revisions enhance clarity and accessibility for an international audience, ensuring that all terms are self-explanatory without requiring prior familiarity with medical abbreviations.

Overall Concerns

8. No scientific or ethical concerns identified. The study shows strong adherence to ethical standards with anonymity and approved protocols.

Response:

We sincerely thank the Editor for recognizing the strong adherence of our study to ethical standards, including the use of anonymity and approved protocols. We greatly appreciate this positive evaluation and encouragement.

Competing Interests

9. Disclosures are complete and present no conflicts impacting study validity.

Response:

We sincerely thank the Editor for acknowledging the completeness of our disclosures and for confirming that no conflicts impact the validity of our study. Additionally, we have included a Competing Interests statement at the end of the manuscript, with the following content: *“The authors have declared that no competing interests exist.”*

English Language Editing

10. The manuscript is written in clear, professional English. Minor stylistic polishing could enhance readability without substantive changes.

Response:

We sincerely thank your positive feedback on the clarity and professionalism of the manuscript’s language. Following your suggestion, we have carried out a comprehensive English language polishing to further improve readability. All modifications have been clearly marked in the submitted tracked‐changes version of the revised manuscript.

**Reviewer #2**

1. This manuscript assesses hepatotoxicity risk factors in HIV-infected MSM with HBV/HCV coinfections in northwest China. The study is thorough, employs suitable statistical methods, and the results support the conclusions. The findings provide valuable evidence for clinicians to optimise ART management in coinfected patients.

Although comprehensive, the study does not fully consider factors such as alcohol consumption or medication adherence, which could influence liver outcomes. Authors should consider elaborating on the limitations, particularly concerning unmeasured confounders such as alcohol consumption, use of traditional medicine, or other hepatotoxic exposures that might influence liver outcomes.

Response:

We sincerely thank the Reviewer for this constructive comment. We fully agree that important factors such as alcohol consumption, medication adherence, and other hepatotoxic exposures may influence liver outcomes. Unfortunately, in our cohort, information on alcohol consumption and use of potentially hepatotoxic medications was not systematically documented in the medical records, and data on medication adherence were incomplete. Consequently, these variables could not be adjusted for in our analysis.

To address this limitation, we have expanded the Discussion section to emphasize that unmeasured confounders, including alcohol consumption, medication adherence, and use of traditional medicines, could affect liver outcomes.

Expanded text in Discussion:

“*Despite its significant scientific value, this study has several limitations. First, the data were derived from a single medical center, which may limit the generalizability of the findings. Future research involving large-scale, multicenter cohort studies is needed to validate these conclusions. Second, although the study provides valuable insights into hepatotoxicity risk factors, information on alcohol consumption and other potential hepatotoxic exposures (such as use of hepatotoxic medications) was not systematically collected in the medical records. These factors could potentially influence liver outcomes and should be considered in future studies to understand the overall risk profile for hepatotoxicity better.*”

2. Analysis for triple infections (e.g., HIV/HBV/HCV coinfection) was excluded.

Response:

We sincerely thank the Reviewer for this important comment. We agree that triple infections (HIV/HBV/HCV) deserve attention. However, in our cohort only six patients presented with triple infections. As stated in the Methods section, “*Patients with triple infections of HIV, HBV, and HCV were excluded from the analysis due to the very small number of such cases (n=6). Specifically, individuals who tested positive for both HBsAg and anti-HCV were excluded.*” The very small sample size limited the statistical power and widened the confidence intervals, which could have affected the accuracy of the statistical results. Therefore, these patients were excluded from the main analysis.

In line with the Reviewer’s suggestion, we have expanded the Discussion section to explicitly describe this limitation and emphasize that the small number of triple infection cases limits the generalizability of our findings. The revised text now reads as follows:

*“Finally, only six cases of HIV, HBV, and HCV triple infections were identified in this cohort. Due to the very limited sample size, statistical power was insufficient and the resulting wide confidence intervals could compromise the accuracy of the findings; therefore, these patients were excluded from the main analysis. Furthermore, due to the limited sample size, the study conclusions may not be generalizable to populations with triple infections, and future studies with larger sample sizes are needed to clarify this issue.”*

3. Some longer sentences might be better divided for clarity, especially in the Discussion section.

Response:

We sincerely thank the Reviewer for this helpful suggestion. In accordance with your advice, we have revised the manuscript by dividing several longer sentences, particularly in the Discussion section, to improve clarity and readability.

4. Could the authors consider incorporating visual summaries, such as a flowchart or simplified diagram of risk factors?

Response:

We thank the reviewer for this valuable suggestion. In response, we have incorporated a visual summary (Figure 4) to present the risk and protective factors for ART-related hepatotoxicity in a simplified diagram. The figure illustrates that HBV/HCV coinfection, older age at ART initiation, and shorter diagnosis-to-treatment interval were identified as risk factors, whereas higher CD4+ counts and higher hemoglobin were protective factors for any grade hepatotoxicity. In addition, HBV coinfection was identified as a risk factor for grade ≥3 hepatotoxicity. We believe that the inclusion of this figure improves clarity and provides readers with a more intuitive understanding of our findings.

Fig 4. Risk and protective factors for ART- related hepatotoxicity. ART, antiretroviral therapy.

5. I recommend providing more practical guidance for clinicians, such as recommending the frequency of liver function tests or screening intervals, to make the findings more actionable.

Response:

We thank the Reviewer for this valuable suggestion. In line with the DHHS Adult and Adolescent HIV Treatment Guidelines (2024), we have revised the Discussion to provide more practical recommendations for clinicians regarding liver function monitoring. The revised discussion section offers the following suggestions to clinicians：

*“From a clinical perspective, our findings highlight the importance of dynamic liver function monitoring in HIV-infected MSM with HBV/HCV coinfections during ART. According to the latest U.S. Department of Health and Human Services (DHHS) Guidelines for the Use of Antiretroviral Agents in Adults and Adolescents with HIV (2024), baseline liver enzyme testing is recommended prior to ART initiation, followed by repeat testing at 2–8 weeks after initiation or regimen change, and every 3–6 months thereafter, with more frequent assessments when using potentially hepatotoxic agents or in high-risk patients. Given the higher hepatotoxicity risk observed in our cohort, we suggest that clinicians consider a more intensive monitoring approach in coinfected patients, particularly during the first year of ART. For example, liver function tests could be conducted every 2-4 weeks initially, with subsequent extension of the interval to 3 months once liver enzymes stabilize. Such an approach may help identify early liver injury, minimize treatment interruptions, and improve the long-term safety of ART in this vulnerable population.”*
